# Supplementary material for: Reductions in Retrieval Competition Predict the Benefit of Repeated Testing
Source: Sci Rep. 2018 Aug 6;8:11714. doi: 10.1038/s41598-018-29686-y (PMC6078947; doi:10.1038/s41598-018-29686-y)
Supplement: Supplementary file 1 — Supplementary Figures [file 41598_2018_29686_MOESM1_ESM.pdf]

Reductions in Retrieval Competition Predict the Benefit of Repeated Testing

Nicole S. Rafidi[1,2*], **Justin C. Hulbert**[2,3], **Paula Pacheco**[2], **and Kenneth A. Norman**[2]

[1]Carnegie Mellon University, Machine Learning Department, Pittsburgh, 15232, USA
[2]Princeton University, Princeton Neuroscience Institute, Princeton, 08544, USA
[2]Bard College, Psychology Program, Annandale-on-Hudson, 12504, USA
[*]nrafidi@cs.cmu.edu

Repeated testing leads to improved long-term memory retention compared to repeated study, but the mechanism underlying this improvement remains controversial. In this work, we test the hypothesis that retrieval practice benefits subsequent recall by reducing competition from related memories. This hypothesis implies that the degree of reduction in competition between retrieval practice attempts should predict subsequent memory for practiced items. To test this prediction, we collected electroencephalography (EEG) data across two sessions. In the first session, participants practiced selectively retrieving exemplars from superordinate semantic categories (high competition), as well as retrieving the names of the superordinate categories from exemplars (low competition). In the second session, participants repeatedly studied and were tested on Swahili-English vocabulary. One week after session two, participants were again tested on the vocabulary. We trained a within-subject classifier on the data from session one to distinguish high and low competition states. We then used this classifier to measure the change in competition across multiple successful retrieval practice attempts in the second session. The degree to which competition decreased for a given vocabulary word predicted whether it was subsequently remembered in the third session. These results are consistent with the hypothesis that repeated testing improves retention by reducing competition.

Supplementary Information

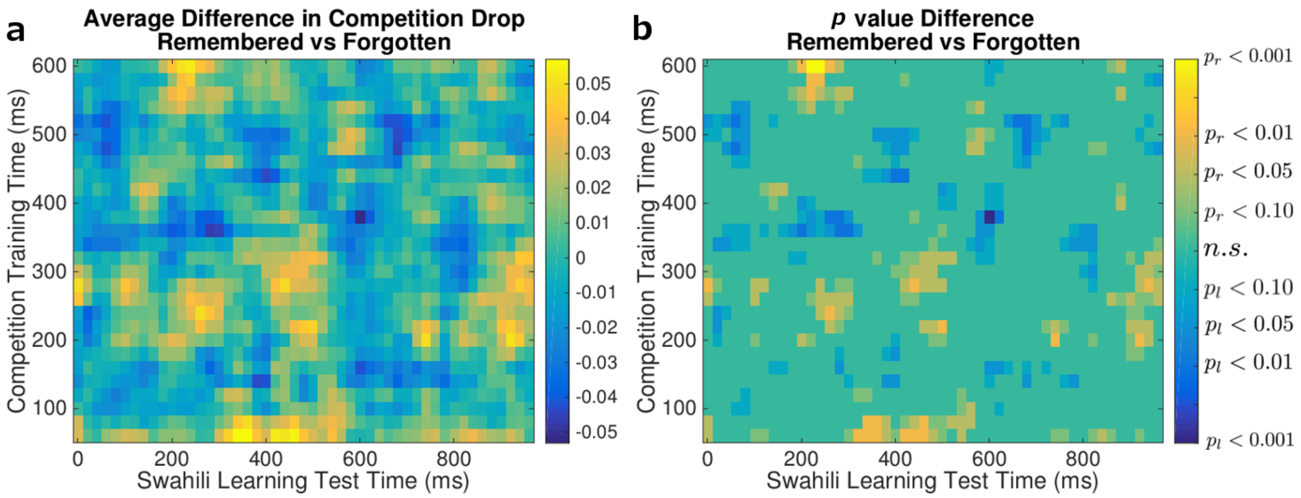

Figure S1. Competition drop over study trials fails to predict subsequent memory. We tested classifiers trained on high-accuracy timepoints from Session 1 on all post-stimulus-onset time points during the Swahili Learning Task in Session 2, this time using only the study trials. This crossing of training and testing time points defines a grid. For each item and each grid square, we computed the *competition drop*: the decrease in competition across successive study trials in the Swahili Learning Task (see text for details). We hypothesized that larger competition drop values in Session 2 would predict better recall in Session 3; we tested all grid squares and corrected for multiple comparisons using a cluster permutation procedure. **a. Average difference in competition drop between remembered and forgotten items.** Yellow indicates that remembered items exhibited a larger competition drop than forgotten items. Blue indicates the opposite. **b. One-tailed t-test result of the difference in competition drop between remembered and forgotten items.** Grid squares for which the drop was larger for remembered items, i.e., where a right-tailed test (p_r) achieved significance at $p_r < 0.10$, are yellow; grid squares for which the drop was larger for forgotten items, i.e., where a left-tailed test (p_l) achieved significance at $p_l < 0.10$, are blue. The right-tailed test corresponds to our hypothesis that a drop in competition is predictive of subsequent memory. We ran a cluster permutation test to see whether any clusters of indvidually-significant grid squares were larger than we would expect due to chance; no clusters survived the permutation test.

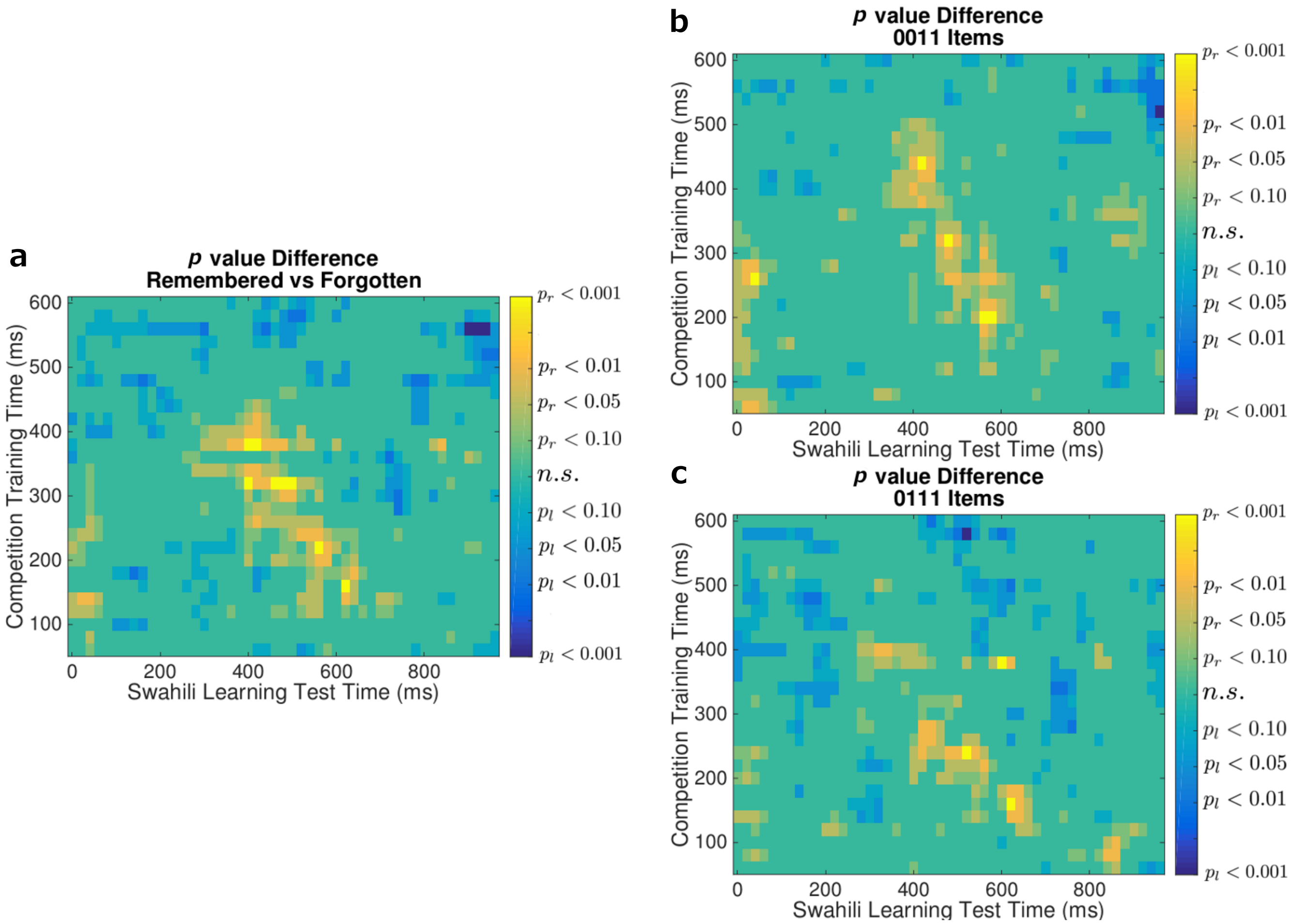

Figure S2. Competition drop analysis with behavior held constant. Comparison of competition drop analysis using all items to that split by behavior during Swahili Learning Task. To evaluate whether the competition drop metric from the EEG data provided addition predictive power over behavior, we re-ran the analysis from Fig. 6 on two distinct subsets of items: items for which subjects were only correct on the last two rounds (0011 items) and items for which subjects were correct on all but the first round (0111 items). **a. One-sided t test results on all trials.** The one-sided t-test result of the difference in competition drop between remembered and forgotten items. Time points for which the drop was larger for remembered items, i.e., where a right-tailed test (p_r) achieved significance, are yellow, whereas time points for which the drop was larger for forgotten items (p_l significant) are blue. **b. Result in subfigure a, computed for 0011 items.** Cluster permutation test significance was $p = 0.01$ for 0011 items. **c. Result in subfigure a, computed for 0111 items.** Cluster permutation test significance was $p = 0.17$ for 0111 items.

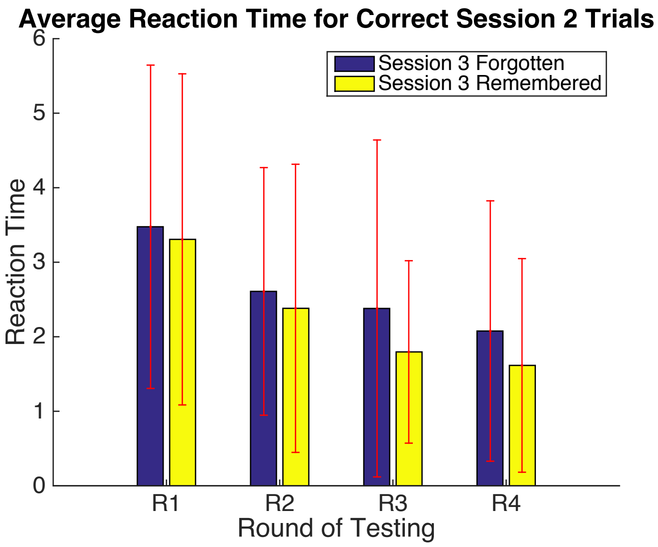

Figure S3. **Average trial reaction time in Session 2, split by Session 3 performance.** Average reaction time for each recall round in Session 2, computed over rounds for which the subject responded correctly. Each bar is the mean over items, pooled across subjects. Error bars show standard deviation over items. Reaction time is measured as the time of first key press after the mandatory 2s wait period (so responding immediately at the end of the wait period corresponds to an RT of zero). Subsequently forgotten items are shown in blue, and subsequently remembered items are shown in yellow.

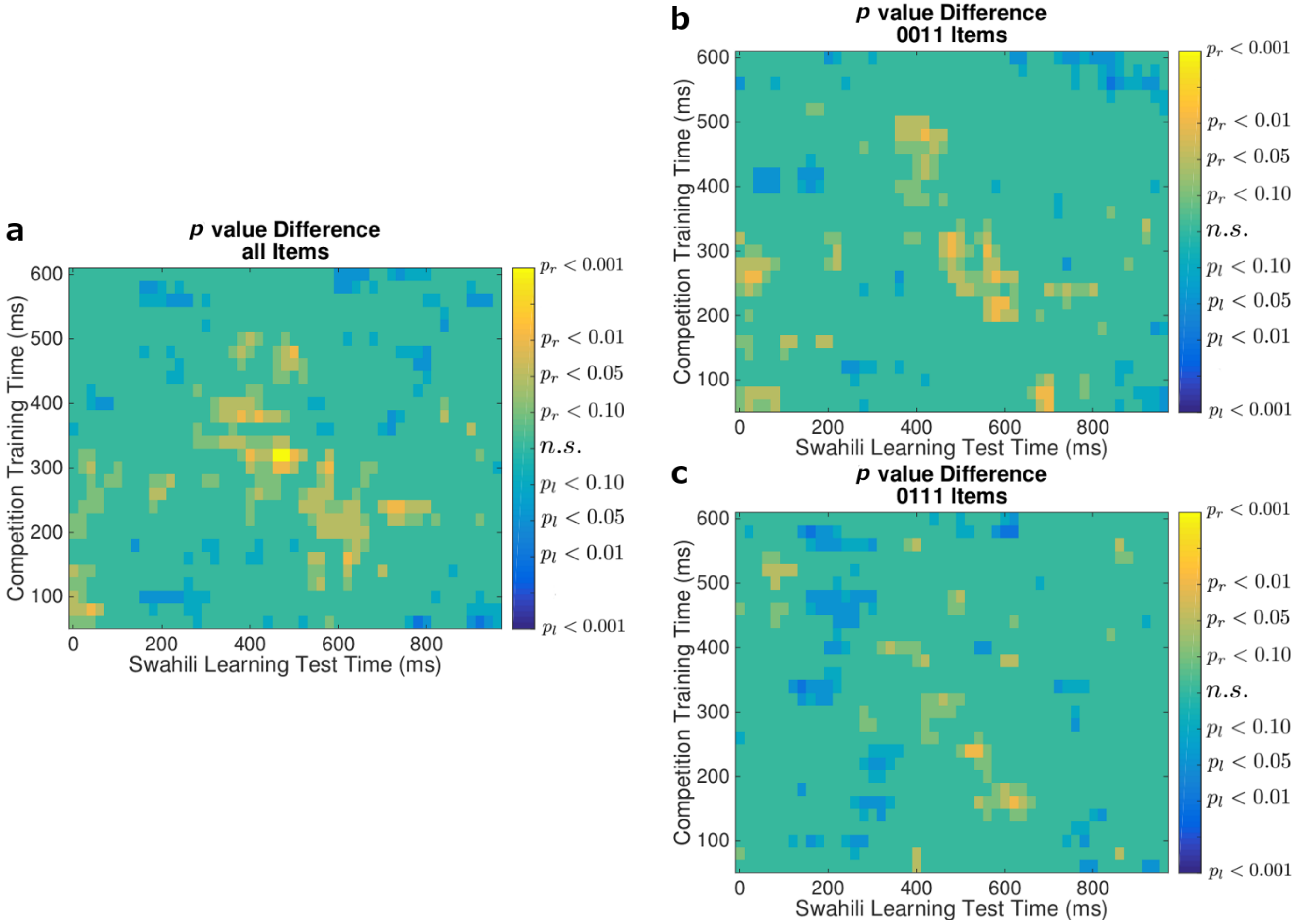

Figure S4. Competition drop analysis with behavior held constant and cross-subject variance mitigated. Results shown in Fig. S2 with within-subject competition drop scores z-scored. **a. One-sided t test results after z-scoring within subject.** Time points for which the drop was larger for remembered items, i.e., where a right-tailed test (p_r) achieved significance, are yellow, whereas time points for which the drop was larger for forgotten items (p_l significant) are blue. Note the similarity to Fig. 6b. **b. Result in subfigure a, computed for 0011 items. c. Result in subfigure a, computed for 0111 items.**

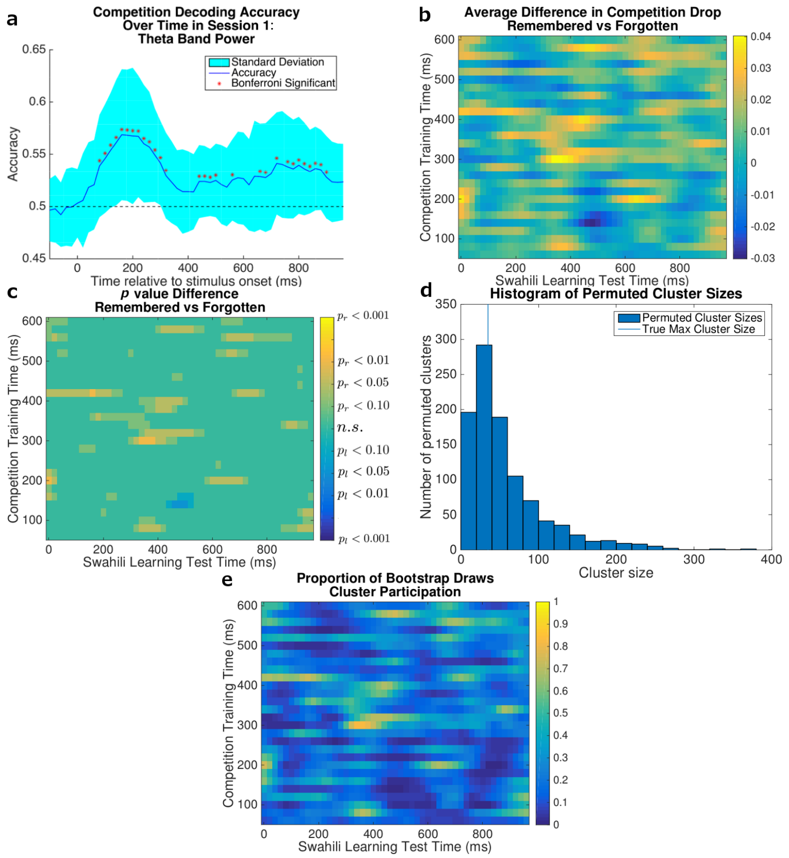

Figure S5. Analyses relating competition drop to subsequent memory, using theta-band power as features. The main analysis summarized in Fig. 4 and Fig. 6, conducted using theta-band power as opposed to voltages. **a. Competition decoding accuracy over time in Session 1** 5-fold cross-validation accuracy at each time point for distinguishing high- and low-competition retrieval states, averaged across subjects. The shaded region gives the standard deviation across the subject means. Chance is 50%. Stars indicate time points that were significant after per-time point permutation test and Bonferroni correction at 0.001 significance threshold. **b. Average difference in competition drop between remembered and forgotten items.** Yellow indicates that remembered items exhibited a larger competition drop than forgotten items. Blue indicates the opposite. **c. One-tailed t-test of the difference in competition drop between remembered and forgotten items.** Grid squares for which the drop was larger for remembered items, i.e., where a right-tailed test (p_r) achieved significance at $p_r < 0.10$, are yellow; grid squares for which the drop was larger for forgotten items, i.e., where a left-tailed test (p_l) achieved significance at $p_l < 0.10$, are blue. The right-tailed test corresponds to our hypothesis that a drop in competition is predictive of memory. No clusters survived multiple corrections. **d. Cluster permutation test histogram** The vertical line indicates the surviving cluster size achieved using the true labels. Each draw in the histogram represents the largest cluster observed in a given permutation of the data. **e. Subject bootstrap results.** Subjects were sampled with replacement; on each draw the cluster analysis was re-run. The color scale on this plot represents the proportion of bootstrap draws in which a point belonged to a significant cluster. A high frequency (yellow) indicates the the point was highly consistent across subjects.

ALCOHOL		APPLIANCE	
BRANDY	CHAMPAGNE	BLENDER	DRYER
GIN	SCOTCH	JUICER	MICROWAVE
TEQUILA	VODKA	REFRIGERATOR	STOVE
WINE	WHISKEY	TOASTER	WASHER
BUILDING		CANDY	
CHURCH	MONUMENT	BUTTERSCOTCH	CARAMEL
MUSEUM	RESTAURANT	GUM	LICORICE
SCHOOL	SKYSCRAPER	LOLLIPOP	MINT
STADIUM	TOWER	SUCKER	TAFFY
DANCE		DISEASE	
DISCO	FOLK	DIABETES	HEPATITIS
JIG	POLKA	MEASLES	MUMPS
SQUARE	TAP	PNEUMONIA	POLIO
TWIST	WALTZ	SMALLPOX	TYPHOID
ELEMENT		EMOTION	
ARGON	BARIUM	ENVY	EXCITEMENT
IODINE	MERCURY	FEAR	LOVE
RADIUM	SULFUR	PITY	SHAME
URANIUM	ZINC	SORROW	TENSION
FABRIC		FLOWER	
BURLAP	DENIM	DAISY	IRIS
FLANNEL	HEMP	LILY	ORCHID
LACE	NYLON	PANSY	POPPY
SATIN	VELVET	TULIP	VIOLET
GEM		INSECT	
AMETHYST	JADE	BEETLE	ANT
ONYX	OPAL	CATERPILLAR	GRASSHOPPER
QUARTZ	SAPPHIRE	LADYBUG	MOSQUITO
TOPAZ	TURQUOISE	TICK	WASP
LANDFORM		PROFESSION	
CRATER	DUNE	ARTIST	DENTIST
GORGE	GULLY	ELECTRICIAN	JOURNALIST
ISLAND	PLATEAU	PILOT	SECRETARY
RAVINE	RIDGE	TEACHER	WRITER
SEASONING		TOOL	
BASIL	CLOVE	DRILL	LADDER
CUMIN	DILL	LEVEL	PLIERS
GINGER	NUTMEG	SCREWDRIVER	SHOVEL
OREGANO	PAPRIKA	VISE	WRENCH
TREE		VEGETABLE	
ASPEN	BIRCH	ASPARAGUS	BROCCOLI
CEDAR	EVERGREEN	EGGPLANT	ONION
HOLLY	PINE	POTATO	RUTABAGA
SPRUCE	WILLOW	SPINACH	ZUCCHINI
WEAPON			
ARROW	BOMB		
CLUB	DAGGER		
POISON	ROCKET		
SWORD	WHIP		

Table S1. Stimuli for the Competition Localizer Experiment.

Swahili	English	Swahili	English
BUU	MAGGOT	THELUJI	SNOW
FARASI	HORSE	MAITI	CORPSE
PUNDA	DONKEY	NYANYA	TOMATO
NDOO	BUCKET	MASHUA	BOAT
GOTI	KNEE	KAPUTULA	SHORTS
ZULIA	CARPET	FUNUNU	RUMOR
ELIMU	SCIENCE	BUSTANI	GARDEN
LESO	SCARF	DAFINA	TREASURE
ADHAMA	HONOR	TUMBILI	MONKEY
GODORO	MATTRESS	VUKE	STEAM
FAGIO	BROOM	SUMU	POISON
CHAKULA	FOOD	KABURI	GRAVE
PIPA	BARREL	TABIBU	DOCTOR
YAI	EGG	HARIRI	SILK
KAA	CRAB	MALKIA	QUEEN
POMBE	BEER	ADUI	ENEMY
PAZIA	CURTAIN	REMBO	ORNAMENT
ZIWA	LAKE	SALA	PRAYER
ROHO	SOUL	USINGIZI	SLEEP
EMBE	MANGO	ZEITUNI	OLIVES
WALI	RICE	MBWA	DOG
GARI	CAR	MLIMA	MOUNTAIN
SURA	FACE	SAMAKI	FISH
MLANGO	DOOR	KOFIA	HAT
MENDE	BEETLE	UPEPO	WIND
KOLEO	SHOVEL	SHAKA	PROBLEM
MTO	PILLOW	JESHI	ARMY
NJIA	ROAD	MAJI	WATER
NYOKA	SNAKE	RINDA	DRESS
UMBU	SIBLING	MAZIWA	MILK

Table S2. Stimuli for the Swahili Learning Task.
